# Safety assessment of *Staphylococcus* phages of the family *Myoviridae* based on complete genome sequences

**DOI:** 10.1038/srep41259

**Published:** 2017-01-24

**Authors:** Zelin Cui, Xiaokui Guo, Ke Dong, Yan Zhang, Qingtian Li, Yongzhang Zhu, Lingbing Zeng, Rong Tang, Li Li

**Affiliations:** 1 Department of Laboratory Medicine, Shanghai General Hospital, Shanghai Jiao Tong University School of Medicine, Shanghai, 200080, China; 2 Department of Immunology and Microbiology, School of Medicine, Shanghai Jiao Tong University, Shanghai, 200025, China; 3 Department of Laboratory Medicine, Ruijin Hospital, Shanghai Jiao Tong University School of Medicine, Shanghai, 200025, China

## Abstract

*Staphylococcus* phages of the *Myoviridae* family have a wide host range and potential applications in phage therapy. In this report, safety assessments of these phages were conducted based on their complete genome sequences. The complete genomes of *Staphylococcus* phages of the *Myoviridae* family were analyzed, and the Open Reading Frame (ORFs) were compared with a pool of virulence and antibiotic resistance genes using the BLAST algorithm. In addition, the lifestyle of the phages (virulent or temperate) was also confirmed using PHACTS. The results showed that all phages were lytic and did not contain resistance or virulence genes based on bioinformatic analyses, excluding the possibility that they could be vectors for the dissemination of these undesirable genes. These findings suggest that the phages are safe at the genome level. The SceD-like transglycosylase, which is a biomarker for vancomycin-intermediate strains, was widely distributed in the phage genomes. Approximately 70% of the ORFs encoded in the phage genomes have unknown functions; therefore, their roles in the antibiotic resistance and virulence of *Staphylococcus aureus* are still unknown and require consideration before use in phage therapy.

Due to the appearance and dissemination of antibiotic-resistant bacteria, concern is increasing that certain bacteria will develop resistance to all known antibiotics, and we will enter a post-antibiotic era. Recently, phage therapy has regained interest among scientists, and some phages have been used in clinical trials with no harmful effects on the trial participants^1^,^2^,^3^.

While phages can be vectors for horizontal gene transfer, several studies have shown that they can also be sources for the transmission of virulence and antibiotic resistance genes among bacteria^4^, thereby accelerating the evolution of virulence and/or antibiotic resistance in bacteria. Phages containing such undesirable genes could be harmful to human health and must be excluded from phage therapy. One study confirmed that Stx production was phage-regulated in stx(2g)-positive strains (Shiga toxin-producing *Escherichia coli* (STEC))^5^. Report also showed that bacteriophages from poultry, cattle and pigs containing antibiotic resistance genes^6^. These studies suggest that bacteriophages could be environmental vectors for the horizontal transfer of virulence and antibiotic resistance genes.

*Staphylococcus* phages of the *Myoviridae* family have been isolated worldwide. Published data show that they have a wide host range and can potentially be used for phage therapy. To date, the complete genome sequences of dozens of *Staphylococcus* phages of the *Myoviridae* family have become available, including phage K, G1, Twort, A5W, Sb-1, ISP, SA5, GH15, JD007, SA11, vB_SauM_Remus, vB_SauM_Romulus, S25-3, S25-4, phiIPLA-RODI, phiIPLA-C1C, phiSA012, Team1, P108, MCE-2014, 812, SA1, Staphy1N, MSA6, 676Z, P4W, and Fi200w^7^,^8^,^9^,^10^,^11^,^12^,^13^,^14^,^15^. These phages can potentially be used for the treatment of infectious diseases caused by *Staphylococcus aureus* in animal models. Phage S25-3 has been shown to prolong life in the silkworm larval infection model and other animal infection models^16^. The *Staphylococcus* phages Romulus and Remus have infected approximately 70% of the tested *S. aureus* strains and display promising lytic activity against these isolates^15^. The *Staphylococcus* phage ISP was shown to be effective against 86% of tested isolates, including relevant methicillin-resistant *S. aureus* (MRSA) strains^7^. These studies demonstrate the potential use of phages for the prevention of infectious diseases caused by *S. aureus*. In the present report, a safety assessment based on complete genome sequences of *Staphylococcus* phages of the *Myoviridae* family was conducted to evaluate their suitability for clinical use.

## Methods

### Complete phage genomes

*Staphylococcus* phages of the *Myoviridae* family, including the previously reported phage JD007, had complete genome sequence data. The complete genome sequences were collected from the NCBI database (http://www.ncbi.nlm.nih.gov/genome/viruses/). All *Staphylococcus* phages belonged to the *Myoviridae* family and were verified by detailed information provided by the NCBI phylogeny system and their morphology. The published papers related to these phages were also screened. The *Staphylococcus* phages were confirmed to belong to the *Myoviridae* family by morphological characterization of the contractile sheath between the head and tail.

### General genomic features of the phages

The complete genome sequences of *Staphylococcus* phages of the *Myoviridae* family were downloaded in FASTA format from NCBI along with their annotation information. The genomes of the phages were re-annotated using RAST, and each ORF and protein domain search was performed using the InterProScan program and CDD^17^,^18^. The phylogeny tree was constructed using MEGA5 with the complete genome sequences based on the neighbour-joining method^19^. The visual alignments of the complete genomes were constructed using the Mauve2.3.1 program^20^.

### Lifestyles of the phages

The lifestyles of phages (virulent or temperate) were predicted using the PHACTS program (http://www.phantome.org/PHACTS/index.htm)^21^. The genome annotation results of each phage were analyzed using PHACTS, and their lifestyle was confirmed. PHACTS utilizes a novel algorithm and a supervised Random Forest classifier to predict whether the lifestyle of a phage is virulent or temperate. The algorithm creates a training set from phages with known lifestyles. PHACTS predictions have been shown to have a 99% precision rate, and PHACTS can also determine the lifestyle of a phage using only genomic data. A current limitation of PHACTS is that a confident lifestyle prediction cannot be made for a small number of phages. This is primarily due to the variability that arises from the random sampling during classifications. If an unknown phage does not have any similarity to phages with known lifestyles in the database, the predictions will be less certain. It is expected that as more phages with known lifestyles are added to the database, the precision rate and sensitivity of the predictions will increase^21^. Additionally, the experimental results of the bacteriophage one step growth curve reported in the corresponding papers were also analyzed to confirm the phage lifestyles.

### Analysis of virulence and antibiotic resistance genes

All annotated phage ORFs were used to search an antibiotic resistance gene database (ARDB, http://ardb.cbcb.umd.edu/)^22^ and a virulence factor database (VFDB, http://www.mgc.ac.cn/VFs/main.htm)^23^. Hits with more than 70% coverage and 30% identity were considered as positive results. Lihong *et al*. improved the infrastructural dataset of VFDB: (i) removed the redundancy introduced by previous releases and generated two hierarchical datasets–one core dataset of experimentally verified virulence factors (VFs) only and another full dataset including all known and predicted VFs. Their efforts enhanced the data quality of the VFDB and promoted the usability of the database in the big data era for the bioinformatic mining of the explosively growing data regarding bacterial VFs^23^. Antibiotic Resistance Genes Database (ARDB)—unifying most of the publicly available information on antibiotic resistance. Each gene and resistance type is annotated with rich information, including resistance profile, mechanism of action, ontology, COG and CDD annotations, as well as external links to sequence and protein databases. ARDB database also supports sequence similarity searches and implements an initial version of a tool for characterizing common mutations that confer antibiotic resistance. The information they provided can be used as compendium of antibiotic resistance factors as well as to identify the resistance genes of newly sequenced genes, genomes, or metagenomes^22^.

### Phylogenetic relationship of the phages

To identify the phylogenetic relationship among *Staphylococcus* phages of the *Myoviridae* family, other *S. aureus* phages, including *Podoviridae* (including *Staphylococcus* phages 44AHJD, 66, GRCS, P68, PT1028, S24-1, and SAP-2) and *Siphoviridae* (including *Staphylococcus* phages 3MRA, 13, 29, 52A, 55, 69, 77, 92, 187, 2638A, DW2, IME-SA4, Ipla7, P954, phi5967PVL, phiSa119, phiSauS-IPLA88, PVL, SA13, SA97, SAP-26, SpaA1, StauST-398-3, StB20, StB20-like, StB27, 3A, 11, 23MRA, 37, 71, 88, 96, B166, B236, CNPH82, Ipla5, JS01, phiBU01, phiETA, phiETA2, phiETA3, phiJB, phiMR11, phiNM1, and phiNM2) (phages’ nucleic acid access numbers refer additional file 1), were chosen to evaluate the phylogenetic relationship using complete genome sequences (http://www.ncbi.nlm.nih.gov/genome/viruses/). The neighbour-joining tree was constructed using MEGA5^19^.

## Results

### General genomic features of *Staphylococcus* phages of the *Myoviridae* family

A total of 22 *Staphylococcus* phages with complete genome sequence data were examined: K, G1, Twort, A5W, Sb-1, ISP, SA5, GH15, JD007, SA11, vB_SauM_Remus, vB_SauM_Romulus, S25-3, S25-4, phiIPLA-RODI, phiIPLA-C1C, phiSA012, Team1, P108, MCE-2014, 812, and SA1^7^,^8^,^9^,^10^,^11^,^12^,^13^,^14^,^15^. The *Staphylococcus* phages Staphy1N, MSA6, 676Z, P4W, and Fi200w, for which only partial genome sequences were available, were excluded from this study. The phages have been isolated worldwide, and all belong to the *Myoviridae* family. As shown in Table 1, the genomes were approximately 127 kb–148 kb in length, contained between 170 and 220 ORFs, and had a GC content of 27–31%. However, the GC content of phage SA1 was 45.83%, which was much higher than other *Staphylococcus* phages of the *Myoviridae* family.

### Lytic or lysogenic analysis

PHACTS, a computational approach used to classify the lifestyle of bacteriophages, was used after the complete genome sequences were annotated and the proteins were converted into the FASTA format. The results showed that the *Staphylococcus* phages K, G1, Twort, A5W, Sb-1, ISP, SA5, GH15, JD007, SA11, vB_SauM_Remus, vB_SauM_Romulus, S25-3, S25-4, phiIPLA-RODI, phiIPLA-C1C, phiSA012, Team1, P108, MCE-2014, 812, and SA1 belong to the *Myoviridae* family and are all lytic phages^7^,^8^,^9^,^10^,^11^,^12^,^13^,^14^,^15^. The previous report shows that phage phiIPLA-RODI and phiIPLA-C1C are lytic phages^11^, our results were consistent with the studies reported previously.

### Genome organization and annotation

**Figure 1:**
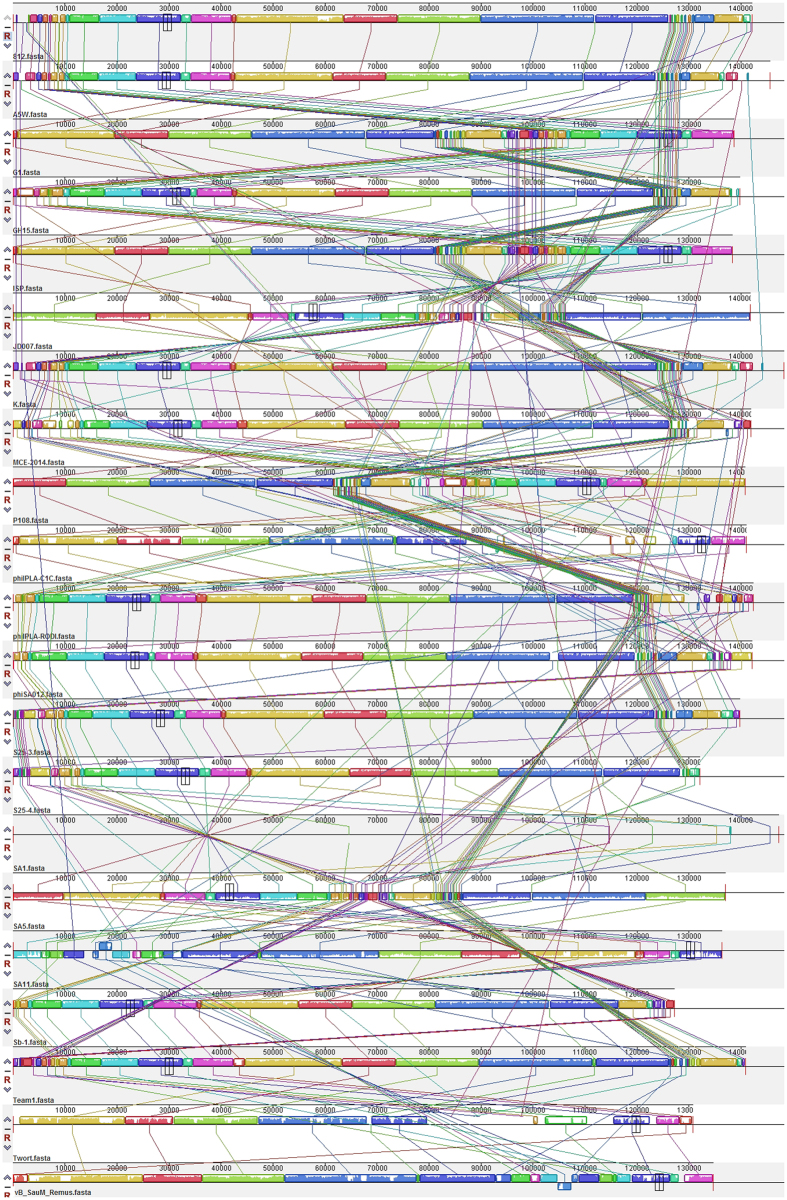
Comparative genomic analysis of *Staphylococcus* phages of the *Myoviridae* family.

The complete genomic sequences of *Staphylococcus* phages of the *Myoviridae* family were compared. As showed in Fig. 1, the genomic structure was chimeric and linear. Several functional modules shown in Table 2 consistently exhibited mosaicism in the genomes, including those involved in packaging, lysis, structure, phage and host interactions, DNA manipulation, and some ORFs with additional functions. High degrees of sequence similarity were evident across the phage sequences, which is unique among *Staphylococcus* phages of the *Myoviridae* family. While, *recombinase* gene was widely existed in the complete genomes of *Staphylococcus* phages of the *Myoviridae* family, what suggest that genomic sequences combination may occur between the homologue sequences during phage infection. The difficulties to knock out *recombinase* gene in these phages may block experimental validation the fully inactivity of Recombinase or confirmation there were no homologue sequences combination occur in the *recombinase* knock-out phages.

### Analysis of virulence and antibiotic resistance genes

All of the predicted ORFs were compared with the sequences in the Antibiotic Resistance Genes Database (ARDB) and the virulence factors database VFDB using the BLASTn algorithm, with the criteria that genes with more than 70% coverage and 30% identity were considered to exhibit positive results. No significant hits based on the ORFs predicted in the complete genomes of the *Staphylococcus* phages of the *Myoviridae* family were obtained.

**Figure 2:**
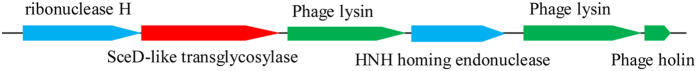
The *SceD*-like transglycosylase gene is widely distributed in the genome of *Staphylococcus* phages of the *Myoviridae* family.

As shown in Fig. 2, the SceD-like transglycosylase, which is a biomarker for vancomycin-intermediate strains^24^, was encoded in the genome of *Staphylococcus* phages K, G1, Twort, A5W, Sb-1, ISP, SA5, GH15, JD007, SA11, vB_SauM_Remus, vB_SauM_Romulus, S25-3, S25-4, phiIPLA-RODI, phiIPLA-C1C, phiSA012, Team1, P108, MCE-2014, and 812^7^,^8^,^9^,^10^,^11^,^12^,^13^,^14^,^15^. Furthermore, approximately 70% of the ORFs were predicted to have unknown functions. It suggested that these ORFs encoding proteins with no similarity functional domains of proteins in InterProScan and CDD database.

### Clustering of *Staphylococcus* phages of the *Myoviridae* family

**Figure 3:**
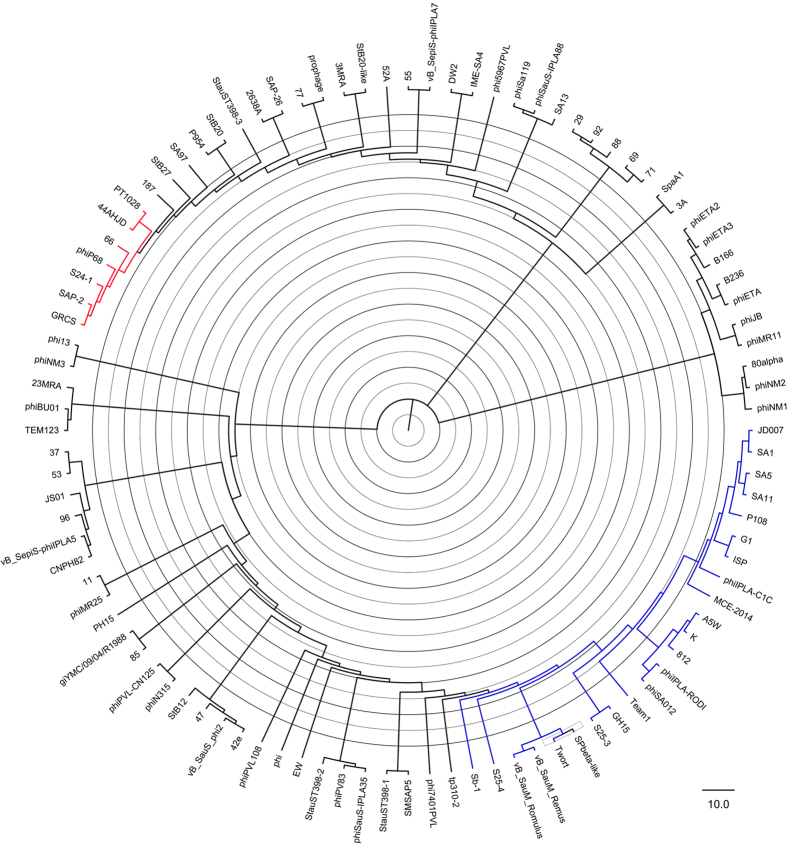
The phylogenetic tree of *Staphylococcus* phages of the *Myoviridae* family. The diagram represents the phylogeny constructed using the complete phage genome sequences via the neighbour-joining method.

The phylogeny tree showed that *Staphylococcus* phages of the *Myoviridae* family clustered in the same branch, based on the alignments of the complete genome sequences. As shown in Fig. 3, the phages K, G1, Twort, A5W, Sb-1, ISP, SA5, GH15, JD007, SA11, vB_SauM_Remus, vB_SauM_Romulus, S25-3, S25-4, phiIPLA-RODI, phiIPLA-C1C, phiSA012, Team1, P108, MCE-2014, 812, and SA1^7^,^8^,^9^,^10^,^11^,^12^,^13^,^14^,^15^ clustered together on the same sub-branch with other *Staphylococcus* phages of the *Myoviridae* family. These results are consistent with the morphology of the phages. It is well known that the proteins such as Helicase, DNA Polymerase, Primase, Large terminase and Major Capsid Protein encoded in the genome of phages are usually chosen to draw phylogenic trees^25^. However, such genes are not always simultaneously existed in the genomes of phages our study enrolled, so it is difficult to cluster phages using these genes when some phages have while others are not. With the development of genome sequencing recently, the complete genome sequences of viruses with high identity were chosen to draw the phylogenic trees with high accuracy.

## Discussions

Phage therapy has become a subject of renewed interest recently, and some phages have been used in clinical trials, including the *Staphylococcus* phages of the *Myoviridae* family. Several studies have evaluated the safety of certain phages in clinical trials. Rhoads *et al*. reported a study in which ulcers were treated for 12 weeks with bacteriophages targeting *Pseudomonas aeruginosa, S. aureus*, and *Escherichia coli* or a saline control^3^. Follow-up continued until week 24, and no adverse events were attributed to the phage therapy. In addition, no significant difference was determined between the test and control groups regarding the frequency of adverse events, rate of healing, or frequency of healing^3^. Unlike normal antibiotics, a bacteriophage is a type of virus that contains genetic material. Therefore, potentially useful phages require full evaluation prior to use in phage therapy. A bacteriophage may be a potential mechanism for the transmission of antibiotic resistance genes or virulence genes among bacteria. A prophage may contribute the pathogenic traits of *Enterococcus feaclis*^26^. Antibiotic resistance genes, including *blaTEM, qnrA, blaCTX-M-1*, and toxin genes, have been found in the DNA of bacteriophages isolated from human faecal samples^27^,^28^. Therefore, safety assessment of phages at the genome level is critically important. First, an analysis should determine whether the bacteriophage carries genes that may accelerate the virulence and antibiotic resistance of bacteria when these genes integrate into their genomes. Second, the ability of the bacteriophage genome that will integrate into the genome of bacteria should be assessed. In this evaluation, it is important and necessary to assess the safety of bacteriophages at the genome level. McCallin *et al*. evaluated the safety of a phage cocktail from one company, and a small volunteer trial did not report an association between adverse effects and oral phage exposure^29^. During that study, the genome sequences of phages were obtained, and bioinformatics analysis revealed that no undesirable genes were present^29^. A safety assessment of the phage at the genome level is an essential step during the evaluation of the suitability of phages for therapeutic applications.

*Staphylococcus* phages of the *Myoviridae* family are potentially useful for phage therapy. Dozens of phages have been shown to protect mice from death after infection with *S. aureus*. Some phages have been used in clinical trials, and no adverse effects have been reported. In this report, our results showed that none of these bacteriophages carried any antibiotic resistance or virulence genes. In addition, all of the studied bacteriophages were virulent and were therefore unable to integrate into the genome of *S. aureus*. Our studies show that *Staphylococcus* phages of the *Myoviridae* family are safe at the genome level. While, as showed in Table 2, *recombinase* was widely distributed in phage GH15, JD007, MCE-2014, phiIPLA-C1C, phiSA012, SA11, Twort. Team1, vB_SauM_Romulus and vB_SauM_Remus, it was reported that Recombinase could recombine homologous genome sequences between bacteriophages or/and its host^30^,^31^; and the SceD-like transglycosylase, which is a biomarker for vancomycin-intermediate strains^24^ is encoded in the genome of *Staphylococcus* phages K, G1, Twort, A5W, Sb-1, ISP, SA5, GH15, JD007, SA11, vB_SauM_Remus, vB_SauM_Romulus, S25-3, S25-4, phiIPLA-RODI, phiIPLA-C1C, phiSA012, Team1, P108, MCE-2014, and 812^7^,^8^,^9^,^10^,^11^,^12^,^13^,^14^,^15^. The expression of this gene during *S. aureus* infection may increase the minimum inhibitory concentration of vancomycin used for the treatment of infections caused by MRSA; furthermore, approximately 70% of the ORFs encoded in the phage genomes have unknown functions, and their potential roles in *S. aureus* antibiotic resistance and virulence are unknown, what’s more, with the development of genome sequencing, acquisition of a complete genome sequence of organism become much more easily, and lots of ORFs encoded proteins with unknown functions^32^. All of the above factors represent a potential genome-level risk for phage therapy. Therefore, the clinical use of these phages should be evaluated comprehensively.

In summary, *Staphylococcus* phages of the *Myoviridae* family have been isolated worldwide. Morphology studies have shown that these phages are contractile between the head and tail. In addition, they have a wide host range and can prevent infectious diseases in animal models, and pre-clinical and clinical trials have not reported any adverse effects. Our results show that *Staphylococcus* phages of the *Myoviridae* family and are lytic and therefore cannot integrate into the genomes of their host. In addition, no antibiotic resistance or virulence genes were present in their genomes, indicating that they are safe at the genome level. In addition, the phages analyzed in this study clustered in the same branch of an evolutionary tree, suggesting that they share a common ancestor.

## Additional Information

**How to cite this article:** Cui, Z. *et al*. Safety assessment of *Staphylococcus* phages of the family *Myoviridae* based on complete genome sequences. *Sci. Rep.* **7**, 41259; doi: 10.1038/srep41259 (2017).

**Publisher's note:** Springer Nature remains neutral with regard to jurisdictional claims in published maps and institutional affiliations.

## Supplementary Material

Supplementary Information

Supplementary Dataset 1

Supplementary Dataset 2

Supplementary Dataset 3

Supplementary Dataset 4

Supplementary Dataset 5

Supplementary Dataset 6

Supplementary Dataset 7

Supplementary Dataset 8

Supplementary Dataset 9

Supplementary Dataset 10

Supplementary Dataset 11

Supplementary Dataset 12

Supplementary Dataset 13

Supplementary Dataset 14

Supplementary Dataset 15

Supplementary Dataset 16

Supplementary Dataset 17

Supplementary Dataset 18

Supplementary Dataset 19

Supplementary Dataset 20

Supplementary Dataset 21

Supplementary Dataset 22

## Figures and Tables

**Table 1:** The *Staphylococcus* phages of the *Myoviridae* family with published complete genome sequences (through June, 2016).

Phages	Country	Size (bp)	GC %	Access No.	Lytic/Lysogenic	Date	Refs
K	USA	127395	30.60	NC_005880	lytic	2004	10
G1	Georgia	138715	30.39	AY954969.1	lytic	2005	33
Twort	Canada	130706	30.26	NC_007021	lytic	2005	33
A5W	Poland	137083	30.47	EU418428.1	lytic	2008	NCBI
Sb-1	Georgia	127188	30.48	HQ163896.1	lytic	2011	34
ISP	Georgia	138339	30.42	FR852584.1	lytic	2011	7
SA5	Georgia	137031	30.42	JX875065	lytic	2012	NCBI
GH15	China	139806	30.23	JQ686190	lytic	2012	14
JD007	China	141836	30.37	JX878671	lytic	2012	13
SA11	Korea	136326	30.04	JX194239	lytic	2012	8
vB_SauM_Remus	Belgium	134643	29.97	JX846612	lytic	2013	15
vB_SauM_Romulus	Belgium	131332	30.01	JX846613	lytic	2013	15
S25-3	Japan	139738	30.22	AB853330	lytic	2013	9
S25-4	Japan	132123	30.31	AB853331	lytic	2013	9
phiIPLA-RODI	Belgium	142348	30.42	KP027446	lytic	2015	11
phiIPLA-C1C	Belgium	140961	27.98	KP027447	lytic	2015	11
phiSA012	Japan	142094	30.31	NC_023573	lytic	2009	35
Team1	Canada	140903	30.33	KC012913.1	lytic	2014	36
P108	China	140807	30.22	KM216423.1	lytic	2014	NCBI
MCE-2014	U.K.	141907	30.38	NC_025416	lytic	2014	37
812	USA	142096	30.40	NC_029080	lytic	2016	NCBI
SA1	USA	147303	45.83	NC_027991	lytic	2015	NCBI

**Table 2:** The functional modules of the ORFs in the genomes of *Staphylococcus* phages of the *Myoviridae* family.

Modules	Functional ORFs	812	A5W	G1	GH15	ISP	JD007	K	MCE-2014	P108	phiI-PLA-C1C	phiIPLA -ROD1	phiSA 012	S25-3	S25-4	SA11	Twort	Sb-1	Team1	SA5	vB_SauM_Romulus	vB_SauM_Remus	SA1
Replication	DNA helicase	+	+	+	+	+	+	+	+	+	+	+	+	+	+	+	+	+	+	+	+	+	+
	DNA polymerase I	+	+	+	+	+	+	+	+	+	+	+	+	+	+	+	+	+	+	+	+	+	+
	DNA primase	+	+	+	+	+	+	+	+		+	+	+	+	+	+	+	+	+	+	+	+	+
	Ribonu-clease H	+	+	+	+	+	+	+	+	+	+	+	+	+	+	+	+	+	+	+	+	+	
Introns	HNH homing endonuc-lease	+	+	+	+	+		+	+		+	+	+			+	+	+	+	+	+	+	+
Lysis	Endolysin	+	+	+	+	+	+	+	+	+	+	+	+	+	+	+	+	+	+	+	+	+	+
	Holin	+	+	+	+	+	+	+	+	+	+	+	+	+	+	+	+	+	+	+	+	+	
Packaging	Terminase, large subunit	+	+	+	+	+	+	+	+	+	+	+	+	+	+	+	+	+	+	+	+	+	+
Structure	Baseplate	+	+	+	+	+	+	+	+	+	+	+	+	+	+	+	+	+	+	+	+	+	+
	Capsid and scaffold	+	+	+	+	+	+	+	+	+	+	+	+	+	+	+	+	+	+	+	+	+	
	Major capsid protein	+	+	+	+	+	+	+	+	+	+	+	+	+	+		+	+	+	+	+	+	+
	Major tail protein	+	+	+	+	+	+	+	+	+		+	+	+	+	+	+	+	+	+	+	+	
	Major tail sheath	+	+	+	+	+	+	+	+	+	+	+	+	+	+	+	+	+	+	+	+	+	
Regulation	DNA transfer protein	+	+	+	+	+	+	+	+	+	+	+		+	+	+		+	+	+	+	+	
	DNA-binding protein	+	+	+	+	+	+	+	+	+	+	+		+	+	+		+	+	+	+	+	
	Integration host factor	+	+	+	+	+	+	+	+	+	+	+	+	+	+	+	+	+	+	+	+	+	
Recomb-ination	Recomb-inase				+		+		+		+		+			+	+		+		+	+	
	Exonuclease	+	+	+	+	+	+	+	+	+	+	+	+	+	+	+	+	+	+	+	+	+	+
	Endon-uclease	+	+	+	+	+	+	+	+	+		+	+	+	+	+	+	+	+	+	+	+	
	RecA protein	+	+	+		+		+		+		+		+	+			+		+			
