# Supplementary material for: Safety assessment of Staphylococcus phages of the family Myoviridae based on complete genome sequences
Source: Sci Rep. 2017 Jan 24;7:41259. doi: 10.1038/srep41259 (PMC5259776; doi:10.1038/srep41259)
Supplement: Supplementary Information [file srep41259-s1.doc]

**Safety assessment of Staphylococcus phages of the family Myoviridae based on complete genome sequences**

*CuiZelin^1*^, GuoXiaokui^2^,Dong Ke^2^,Zhang Yan^2^, Li Qingtian^3^,Zhu Yongzhang^2^,Zeng Lingbing^2^,Tang Rong^1^, Li Li^1^*^*^

(^1^*Department of Laboratory Medicine, Shanghai General Hospital, Shanghai Jiao Tong University School of Medicine, Shanghai 200080, China*)

(^2^*Department of Immunology and Microbiology*, *School of Medicine*, *Shanghai Jiao Tong University*, *Shanghai*,200025 *China*)

(^3^*Department of Laboratory Medicine, Ruijin Hospital, Shanghai Jiao Tong University School of Medicine, Shanghai,*200025 *China*)

**Corresponding author. Tel:+86-21-63240090-4603*

E-mail address:*Li Li [annylish@126.com](mailto:annylish@126.com), address: 6^th^ floor, 3# building, 100# Haining Road, Shanghai, China, 200080;

Guo Xiaokui [xkguo@shsmu.edu.cn](mailto:xkguo@shsmu.edu.cn);

Dong Ke [kiwidong@126.com](mailto:kiwidong@126.com);

Zhang Yan [zhangyan007236@126.com](mailto:zhangyan007236@126.com);

Li Qingtian [qingtianli@sjtu.edu.cn](mailto:qingtianli@sjtu.edu.cn);

Zhu Yongzhang [yzhzhu@126.com](mailto:yzhzhu@126.com);

Zeng Lingbing [lingbing_zeng@163.com](mailto:lingbing_zeng@163.com);

Tang Rong [tangrong5059@126.com](mailto:tangrong5059@126.com);

*Cui Zelin [czl@sjtu.edu.cn](mailto:czl@sjtu.edu.cn); address:6^th^ floor, 3# building, 100# Haining Road, Shanghai, China, 200080.

| **Staphylococcus phages involved in this study** | **Family** |
| --- | --- |
| gi|985759046|ref|NC_029080.1| Staphylococcus phage 812, complete genome | Myoviridae |
| gi|594138638|gb|EU418428.2| Staphylococcus phage A5W, complete genome | Myoviridae |
| gi|66394949|ref|NC_007066.1| Staphylococcus phage G1, complete genome | Myoviridae |
| gi|418487912|ref|NC_019448.1| Staphylococcus phage GH15, complete genome | Myoviridae |
| gi|345134215|emb|FR852584.1| Staphylococcus phage ISP complete genome | Myoviridae |
| gi|428782997|ref|NC_019726.1| Staphylococcus phage JD007, complete genome | Myoviridae |
| gi|657184119|ref|NC_005880.2| Staphylococcus phage K, complete genome | Myoviridae |
| gi|725948655|ref|NC_025416.1| Staphylococcus phage MCE-2014, complete genome | Myoviridae |
| gi|712912451|ref|NC_025426.1| Staphylococcus phage P108, complete genome | Myoviridae |
| gi|971761109|ref|NC_028962.1| Staphylococcus phage phiIPLA-C1C, complete genome | Myoviridae |
| gi|971741276|ref|NC_028765.1| Staphylococcus phage phiIPLA-RODI, complete genome | Myoviridae |
| gi|589286664|ref|NC_023573.1| Staphylococcus phage phiSA012 DNA, complete genome | Myoviridae |
| gi|561133277|ref|NC_022920.1| Staphylococcus phage S25-3 DNA, complete genome | Myoviridae |
| gi|561133064|ref|NC_022918.1| Staphylococcus phage S25-4 DNA, complete genome | Myoviridae |
| gi|937456792|ref|NC_027991.1| Staphylococcus phage SA1, complete genome | Myoviridae |
| gi|410808913|gb|JX875065.1| Staphylococcus phage SA5, complete genome | Myoviridae |
| gi|422935575|ref|NC_019511.1| Staphylococcus phage SA11, complete genome | Myoviridae |
| gi|564292828|ref|NC_023009.1| Staphylococcus phage Sb-1, complete genome | Myoviridae |
| gi|985761090|ref|NC_029119.1| Staphylococcus phage SPbeta-like, complete genome | Myoviridae |
| gi|725948879|ref|NC_025417.1| Staphylococcus phage Team1, complete genome | Myoviridae |
| gi|66391255|ref|NC_007021.1| Staphylococcus phage Twort, complete genome | Myoviridae |
| gi|530787450|ref|NC_022090.1| Staphylococcus phage vB_SauM_Remus, complete genome | Myoviridae |
| gi|472437772|ref|NC_020877.1| Staphylococcus phage vB_SauM_Romulus, complete genome | Myoviridae |
| gi|29565721|ref|NC_004678.1| Staphylococcus phage 44AHJD, complete genome | Podoviridae |
| gi|66395187|ref|NC_007046.1| Staphylococcus phage 66, complete genome | Podoviridae |
| gi|584590862|gb|KJ210330.1| Staphylococcus phage GRCS, complete genome | Podoviridae |
| gi|29565743|ref|NC_004679.1| Staphylococcus phage phiP68, complete genome | Podoviridae |
| gi|66395164|ref|NC_007045.1| Staphylococcus phage PT1028, complete genome | Podoviridae |
| gi|371671319|ref|NC_016565.1| Staphylococcus phage S24-1, complete genome | Podoviridae |
| gi|157738587|ref|NC_009875.1| Staphylococcus phage SAP-2, complete genome | Podoviridae |
| gi|66395508|ref|NC_007052.1| Staphylococcus phage 42e, complete genome | Siphoviridae |
| gi|66395656|ref|NC_007054.1| Staphylococcus phage 47, complete genome | Siphoviridae |
| gi|66395370|ref|NC_007049.1| Staphylococcus phage 53, complete genome | Siphoviridae |
| gi|148717842|ref|NC_009526.1| Staphylococcus phage 80alpha, complete genome | Siphoviridae |
| gi|66394870|ref|NC_007050.1| Staphylococcus phage 85, complete genome | Siphoviridae |
| gi|66395807|ref|NC_007056.1| Staphylococcus phage EW, complete genome | Siphoviridae |
| gi|215401108|ref|NC_011612.1| Staphylococcus phage phiSauS-IPLA35, complete genome | Siphoviridae |
| gi|119967833|ref|NC_008723.1| Staphylococcus phage PH15, complete genome | Siphoviridae |
| gi|29028617|ref|NC_004616.1| Staphylococcus prophage phi 12, complete genome | Siphoviridae |
| gi|448244641|ref|NC_020199.1| Staphylococcus phage phi7401PVL DNA, complete genome | Siphoviridae |
| gi|189427122|ref|NC_010808.1| Staphylococcus phage phiMR25, complete genome | Siphoviridae |
| gi|30043925|ref|NC_004740.1| Staphylococcus prophage phiN315, complete genome | Siphoviridae |
| gi|118725053|ref|NC_008617.1| Staphylococcus phage phiNM3, complete genome | Siphoviridae |
| gi|119443652|ref|NC_008689.1| Staphylococcus phage phiPVL108, complete genome | Siphoviridae |
| gi|239507361|ref|NC_012784.1| Staphylococcus phage phiPVL-CN125, complete genome | Siphoviridae |
| gi|422935796|ref|NC_019513.1| Staphylococcus phage SMSAP5, complete genome | Siphoviridae |
| gi|509140090|ref|NC_021326.1| Staphylococcus phage StauST398-1, complete genome | Siphoviridae |
| gi|509139878|ref|NC_021323.1| Staphylococcus phage StauST398-2, complete genome | Siphoviridae |
| gi|939535479|ref|NC_020490.2| Staphylococcus phage StB12, complete genome | Siphoviridae |
| gi|388570316|ref|NC_017968.1| Staphylococcus phage TEM123, complete genome | Siphoviridae |
| gi|939489505|ref|NC_009762.3| Staphylococcus phage tp310-2, complete sequence | Siphoviridae |
| gi|971750132|ref|NC_028862.1| Staphylococcus phage vB_SauS_phi2, complete genome | Siphoviridae |
| gi|557308055|ref|NC_022758.1| Staphylococcus phage YMC/09/04/R1988, complete genome | Siphoviridae |
| gi|9635677|ref|NC_002486.1| Staphylococcus prophage phiPV83, complete genome | Siphoviridae |
| gi|971755460|ref|NC_028917.1| Staphylococcus phage 3MRA, complete genome | Siphoviridae |
| gi|29028667|ref|NC_004617.1| Staphylococcus prophage phi 13, complete genome | Siphoviridae |
| gi|66396191|ref|NC_007061.1| Staphylococcus phage 29, complete genome | Siphoviridae |
| gi|66396267|ref|NC_007062.1| Staphylococcus phage 52A, complete genome | Siphoviridae |
| gi|66396113|ref|NC_007060.1| Staphylococcus phage 55, complete genome | Siphoviridae |
| gi|66395293|ref|NC_007048.1| Staphylococcus phage 69, complete genome | Siphoviridae |
| gi|41189515|ref|NC_005356.1| Staphylococcus phage 77, complete genome | Siphoviridae |
| gi|66396406|ref|NC_007064.1| Staphylococcus phage 92, complete genome | Siphoviridae |
| gi|66395215|ref|NC_007047.1| Staphylococcus phage 187, complete genome | Siphoviridae |
| gi|66395450|ref|NC_007051.1| Staphylococcus phage 2638A, complete genome | Siphoviridae |
| gi|658608029|ref|NC_024391.1| Staphylococcus phage DW2, complete genome | Siphoviridae |
| gi|971766686|ref|NC_029025.1| Staphylococcus phage IME-SA4, complete genome | Siphoviridae |
| gi|399529119|ref|NC_018284.1| Staphylococcus phage vB_SepiS-phiIPLA7, complete genome | Siphoviridae |
| gi|257136356|ref|NC_013195.1| Staphylococcus phage P954, complete genome | Siphoviridae |
| gi|431810244|ref|NC_019921.1| Staphylococcus phage phi5967PVL DNA, complete genome | Siphoviridae |
| gi|725915949|ref|NC_025460.1| Staphylococcus phage phiSa119, complete genome | Siphoviridae |
| gi|215401171|ref|NC_011614.1| Staphylococcus phage phiSauS-IPLA88, complete genome | Siphoviridae |
| gi|9635165|ref|NC_002321.1| Staphylococcus prophage PVL, complete genome | Siphoviridae |
| gi|526244872|ref|NC_021863.1| Staphylococcus phage SA13, complete genome | Siphoviridae |
| gi|971765337|ref|NC_029010.1| Staphylococcus phage SA97, complete genome | Siphoviridae |
| gi|304443238|ref|NC_014460.1| Staphylococcus phage SAP-26, complete genome | Siphoviridae |
| gi|399498862|ref|NC_018277.1| Staphylococcus phage SpaA1, complete genome | Siphoviridae |
| gi|509140939|ref|NC_021332.1| Staphylococcus phage StauST398-3, complete genome | Siphoviridae |
| gi|431809730|ref|NC_019915.1| Staphylococcus phage StB20, complete genome | Siphoviridae |
| gi|971746468|ref|NC_028821.1| Staphylococcus phage StB20-like, complete genome | Siphoviridae |
| gi|431809676|ref|NC_019914.1| Staphylococcus phage StB27, complete genome | Siphoviridae |
| gi|66395588|ref|NC_007053.1| Staphylococcus phage 3A, complete genome | Siphoviridae |
| gi|29028563|ref|NC_004615.1| Staphylococcus phage 11, complete genome | Siphoviridae |
| gi|971742259|ref|NC_028775.1| Staphylococcus phage 23MRA, complete genome | Siphoviridae |
| gi|66395729|ref|NC_007055.1| Staphylococcus phage 37, complete genome | Siphoviridae |
| gi|66396040|ref|NC_007059.1| Staphylococcus phage 71, complete genome | Siphoviridae |
| gi|66396333|ref|NC_007063.1| Staphylococcus phage 88, complete genome | Siphoviridae |
| gi|66395885|ref|NC_007057.1| Staphylococcus phage 96, complete genome | Siphoviridae |
| gi|971749870|ref|NC_028859.1| Staphylococcus phage B166, complete genome | Siphoviridae |
| gi|971755286|ref|NC_028915.1| Staphylococcus phage B236, complete genome | Siphoviridae |
| gi|119953678|ref|NC_008722.1| Staphylococcus phage CNPH82, complete genome | Siphoviridae |
| gi|399528895|ref|NC_018281.1| Staphylococcus phage vB_SepiS-phiIPLA5, complete genome | Siphoviridae |
| gi|538397808|ref|NC_021773.2| Staphylococcus phage JS01, complete genome | Siphoviridae |
| gi|744692776|ref|NC_026016.1| Staphylococcus phage phiBU01, complete genome | Siphoviridae |
| gi|17426228|ref|NC_003288.1| Staphylococcus phage phiETA, complete genome | Siphoviridae |
| gi|122891714|ref|NC_008798.1| Staphylococcus phage phiETA2, complete genome | Siphoviridae |
| gi|122891784|ref|NC_008799.1| Staphylococcus phage phiETA3, complete genome | Siphoviridae |
| gi|966198842|ref|NC_028669.1| Staphylococcus phage phiJB, complete genome | Siphoviridae |
| gi|162290107|ref|NC_010147.1| Staphylococcus phage phiMR11, complete genome | Siphoviridae |
| gi|118430724|ref|NC_008583.1| Staphylococcus phage phiNM1, complete genome | Siphoviridae |
| gi|971755109|ref|NC_028913.1| Staphylococcus aureus phage phiNM2, complete genome | Siphoviridae |
